# Thinking Brag

**Published:** 1861-11

**Authors:** 


**THINKING BRAG.**—"It don't do for me even to think brag," said a worthy matron of the Society of Friends, whose long experience of the uncertain tenure of earthly goods had deeply engraven on her mind the pertinent expression of the sacred volume, that we "know not what a day may bring forth." Every year of her long and serene pilgrimage had but added a new demonstration of the wisdom of the same blessed book, in its injunction upon all to "walk softly;" to avoid being "puffed up" by any amount of worldly prosperity; to feel ready, at a moment's warning, to go down into the valley of humility without a murmur, whenever the providences of a loving heavenly Father seemed to point that way. This it is to "walk humbly," and to possess that "lowliness" of heart, which secures the "promise of the life that now is, and of that which is to come." Such a humility begets trust and that habit of serenity which so generally characterizes that excellent people, and which will soon become the ruling element in the character of any individual who, under all prosperities, fears "even to think brag;" a serenity which softens ecstasy, which "eases up" the calamities of life, and so regulates its pulses, that, beating uniformly, the human machine works without a shock or a jar, and runs healthfully to a calm old age.

